# Impact of Elevated Brain IL-6 in Transgenic Mice on the Behavioral and Neurochemical Consequences of Chronic Alcohol Exposure

**DOI:** 10.3390/cells12182306

**Published:** 2023-09-19

**Authors:** Donna L. Gruol, Delilah Calderon, Salvador Huitron-Resendiz, Chelsea Cates-Gatto, Amanda J. Roberts

**Affiliations:** 1 Neuroscience Department, The Scripps Research Institute, La Jolla, CA 92037, USA; 2 Animal Models Core Facility, The Scripps Research Institute, La Jolla, CA 92037, USAaroberts@scripps.edu (A.J.R.)

**Keywords:** neuroimmune, synaptic transmission, alcohol drinking, depressive-like behavior, STAT3, p42/44MAPK, GABA_A_R subunits

## Abstract

Alcohol consumption activates the neuroimmune system of the brain, a system in which brain astrocytes and microglia play dominant roles. These glial cells normally produce low levels of neuroimmune factors, which are important signaling factors and regulators of brain function. Alcohol activation of the neuroimmune system is known to dysregulate the production of neuroimmune factors, such as the cytokine IL-6, thereby changing the neuroimmune status of the brain, which could impact the actions of alcohol. The consequences of neuroimmune–alcohol interactions are not fully known. In the current studies we investigated this issue in transgenic (TG) mice with altered neuroimmune status relative to IL-6. The TG mice express elevated levels of astrocyte-produced IL-6, a condition known to occur with alcohol exposure. Standard behavioral tests of alcohol drinking and negative affect/emotionality were carried out in homozygous and heterozygous TG mice and control mice to assess the impact of neuroimmune status on the actions of chronic intermittent alcohol (ethanol) (CIE) exposure on these behaviors. The expressions of signal transduction and synaptic proteins were also assessed by Western blot to identify the impact of alcohol–neuroimmune interactions on brain neurochemistry. The results from these studies show that neuroimmune status with respect to IL-6 significantly impacts the effects of alcohol on multiple levels.

## 1. Introduction

Recent studies have identified the neuroimmune system of the brain as an important target of alcohol and a significant contributor to the neurochemical and physiological effects of alcohol on the brain, although much remains to be discovered about alcohol–neuroimmune interactions (reviewed in [1,2,3,4]). The neuroimmune system is an important homeostatic regulator of brain function and development, a role that is accomplished through the production and secretion of signaling factors, referred to as neuroimmune factors. These factors are regulators of gene expression, neurochemical pathways, neuronal excitability, synaptic function and, consequently, behavior. The primary sources of these neuroimmune factors are the brain astrocytes and microglia, the key cellular constituents of the brain neuroimmune system, although neurons and other brain cell types can also produce neuroimmune factors depending on the circumstance (e.g., [5,6,7]). As homeostatic regulators, astrocytes and microglia constitutively produce low levels of neuroimmune factors. However, these cell types are very sensitive to brain status and respond to adverse conditions such as excessive alcohol use by elevating the production of neuroimmune factors, which then serve as important contributors to recovery and repair processes. However, if the elevated production becomes dysregulated, instead of contributing to the recovery and repair of the neuroimmune factors, the neuroimmune factors can contribute to brain damage and impaired cognitive function [8,9].

The chronic activation of the neuroimmune system and elevated levels of neuroimmune factors are characteristic of alcohol use disorders (AUD) and other CNS conditions that are associated with impaired brain function such as neurodegenerative diseases, major depression, maladaptive stress responses, neuropsychiatric disorders, autism, injury, and even aging [1,2,10,11,12,13,14,15,16,17,18,19]. Some of these conditions are often co-morbid with AUD.

Multiple neuroimmune factors can be produced by the activation of the neuroimmune system, including IL-6, the focus of the current studies, which investigate the consequences of elevated levels of IL-6 in the brain and IL-6–alcohol interactions. Alcohol exposure is known to increase the levels of IL-6 mRNA or proteins in the brain, an effect that can vary with the level of and time after alcohol exposure, route of administration, brain region, and behavioral context [20,21,22,23,24,25,26]. Altered levels of IL-6 signal transduction partners, which are responsible for the bioactivity of IL-6 and are also altered with alcohol exposure [27,28,29,30]. 

Elevated brain levels of IL-6 can have significant consequences for brain function and behavior. For example, elevated brain levels of IL-6 are associated with depressive-like behaviors, emotionality, and impaired auditory fear learning [31,32,33]. Studies of mice lacking IL-6 also indicate that IL-6 is involved in emotionality [34]. Abnormal levels of IL-6 (elevated or reduced) negatively affect the hippocampal memory performance [5,35,36,37,38,39,40] and influence alcohol consumption [28,41]. Studies, primarily in vitro, have shown that IL-6 can alter neuronal excitability, synaptic transmission, and synaptic plasticity [42,43,44,45,46,47,48,49], which are actions that presumably underlie the effects of IL-6 on brain function and, consequently, behavior. 

Our previous studies using a transgenic model, in which IL-6 levels are persistently elevated in the brain through increased astrocyte expression, show that the transgenic mice exhibit behaviors associated with negative affect/emotionality [50], consistent with studies where IL-6 levels in the brain were increased using other methods. Alcohol exposure/abstinence is also associated with behaviors involving negative affect/emotionality and impaired memory performance [51,52,53]. For example, in rodent studies, binge alcohol drinking, a common pattern of excessive alcohol consumption in humans, causes increased emotionality after the cessation of alcohol drinking (e.g., elevated anxiety- and depressive-like behavior) [54]. Increased emotionality [55] and impairment in learning and memory were also observed after the cessation of chronic alcohol exposure [56]. 

Our previous studies also showed that alcohol and IL-6 target many of the same synaptic and molecular mechanisms and that interactions can occur between alcohol and IL-6 actions (e.g., [30,57,58]). However, it is unknown if these IL-6–alcohol interactions have consequences for behavior. In the current studies, we examined this possibility in homozygous (+/+) and heterozygous (+/−) IL-6 transgenic (TG) mice, with wildtype mice (littermates) used as controls. The TG mice were used as a model for the neuroimmune status of the brain with respect to IL-6 in subjects who persistently consume high levels of alcohol, which is known to increase IL-6 levels in the brain. In the first series of experiments, the effects of chronic intermittent alcohol exposure (CIE), which is known to increase alcohol drinking, on two-bottle choice drinking (2BC) was investigated. Following the alcohol drinking studies, the mice were subjected to behavioral tests designed to examine negative affect/emotionality. The behavioral tests included the light/dark transfer, digging, open field, tail flick, and forced swim tests, all of which are known to be sensitive to alcohol exposure/abstinence [51,59,60]. Some of these behaviors have been shown to be altered by the transgene expression in alcohol-naive IL-6 TG mice [50]. Finally, the mice were euthanized and the neuroimmune and neurochemical status of their brain (the hippocampus was studied) was assessed by the determination of the levels of proteins known to be affected by other alcohol exposure paradigms in the TG mice. Determining neuroimmune status involved measurements of the levels of IL-6, IL-6 signal transduction partners, and TNF-alpha, which show regulatory interactions with the IL-6 signaling system [28,61,62,63,64]. Furthermore, this involved measurements of synaptic proteins. The results show that neuroimmune status with respect to IL-6 impacts the effects of 2BC and CIE on behavior, brain neuroimmunity, and neurochemistry, supporting the idea that the alcohol-induced expression of IL-6 can be an important factor in the effects of alcohol on the brain and behavior.

## 2. Materials and Methods

### 2.1. Transgenic Mice

A total of 15 +/+TG (6 males, 9 females), 50 +/−TG (31 males, 17 females), and 38 WT (17 males, 25 females) mice at 3 months of age were used for these studies. The construction of the TG mice (167 line) has been previously described [65]. The mice were constructed on an SJL background and then backcrossed onto C57BL/6J. The mice are congenic and have been maintained on the C57BL/6J background for many years. The three mouse genotypes (+/−TG, +/+TG, and WT littermates) were obtained by breeding +/−TG female mice with +/−TG male mice. The mice were genotyped commercially using tail DNA analysis (Transnetyx, Cordova, TN, USA) and in the lab using standard PCR methods. Post-mortem protein analyses were also used to confirm +/−TG and +/+TG genotypes based on our previous studies that examined the expression of proteins expected to differ between genotypes (e.g., levels of IL-6, pSTAT3, and GFAP) (e.g., [30,66]) . The mice were group housed at all times except during the two-hour drinking sessions.

### 2.2. Two-Bottle Choice Drinking-Chronic Intermittent Alcohol Exposure Protocol (2BC-CIE)

**Figure 1:**
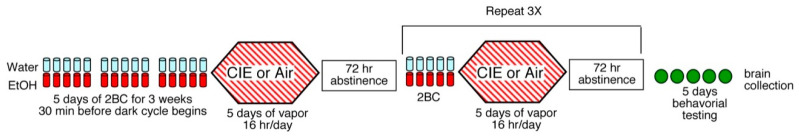
Diagram of alcohol exposure protocol.

In this protocol (Figure 1), for 15 days (5 days per week for 3 weeks), 30 min before the lights were turned off, mice were singly housed for two hours with access to two drinking tubes, one containing 15% alcohol (ethanol) and the other containing water (i.e., a two-bottle choice; 2BC). Alcohol and water consumption during these 2-h periods were recorded. Following this baseline period, mice were divided, based on equal alcohol and water consumptions, into two balanced groups per genotype per sex. The mice were subsequently exposed to intermittent alcohol (ethanol) vapor or air as the control. The vapor groups were injected with 1.75 g/kg alcohol + 68.1 mg/kg pyrazole (alcohol dehydrogenase inhibitor) and placed in the chambers to receive intermittent vapor for 4 days (16 h vapor on, 8 h off). The alcohol vapor chambers (La Jolla Alcohol Research, Inc., La Jolla, CA, USA) could house up to 5 mice per chamber. Identical chambers into which air was pumped were used for control chambers. On the third day of vapor, immediately following a 16 h bout of vapor, mice were removed from the chamber and tail blood was obtained to determine blood alcohol levels (BAL). Target blood alcohol levels were 175–250 mg/dL. Following the fourth day of exposure, mice were allowed 72 h of undisturbed time, followed by 5 days of 2-h access to bottles containing 15% alcohol and water to measure alcohol consumption following vapor chamber exposure. The control mice were injected with 68.1 mg/kg pyrazole in saline for the same periods as the vapor groups and then received 2BC testing at the same time as the vapor groups. The vapor/control exposure and 5 days of 2-bottle choice testing was repeated for a total of 4 cycles of vapor and 2BC testing. The mouse CIE model, originally developed by Howard Becker and colleagues [67] and adopted by the Roberts lab [30,68,69], is widely accepted in the alcohol community.

Mean body weights of the mice were measured at the end of the treatment period and were higher in males than females, but there was no genotype x treatment x sex difference, and weights for females and males were combined for presentation purposes. There was no significant genotype (*F*(2,99) = 2.3, *p* = 0.10), treatment effect (*F*(1,99) = 1.7, *p* = 0.19) or genotype x treatment interaction (*F*(2,99) = 0.5, *p* = 0.58) for animal weight. Mean body weights in grams were 24.6 ± 1.0 (n = 18) and 24.6 ± 0.8 (n = 42) for control and vapor-treated WT mice, respectively, 25.4 ± 0.9 (n = 24) and 27.2± 0.7 (n = 24) for control and vapor-treated +/−TG mice, respectively, and 23.5 ± 1.5 (n = 8) and 25.3 ± 1.6 (n = 24) for control and vapor-treated +/+TG mice, respectively.

### 2.3. Behavior Tests for Negative Affect/Emotionality

Mice were examined for negative affect/emotionality in a series of behavioral tests, as described previously, [50] beginning 3 days after removal from the vapor/air chambers. The sequence for testing is shown in Table 1. In some behavioral tests, outliers were observed and confirmed by the Inter-Quartile Range Method. The outliers were not included in the data analysis.

#### 2.3.1. Light/Dark Transfer Test

This test has been used to assess anxiety-like behavior and exploratory drive [70,71]. The apparatus used consists of a rectangular Plexiglas box divided by a partition into two environments, one highly illuminated from above by a 60 W light source (400–600 lux) (28.5 × 27 × 26.5 cm) and one dark (14.5 × 27 × 26.5 cm; 8–16 lux). An opening (7.5 × 7.5 cm) at the floor level in the center of the partition connects the two compartments. To start the 5-min test, the mice were placed in the dark compartment and the time spent in each compartment and the number of dark-to-light transitions were recorded. A greater amount of time spent in the light compartment and/or a greater number of dark-to-light transitions are indicative of decreased anxiety-like behavior. For this test, there were two outliers for time in the light compartment (males: +/−TG 2BC and +/−TG 2BC-CIE). One of these mice was also an outlier for the number of transitions (+/−TG 2BC). 

#### 2.3.2. Digging Test

Digging is a normal rodent behavior that is considered to reflect response to novelty [72] and possibly irritability [55]. In this test, mice were placed individually in a standard mouse cage containing bedding 5 cm in depth. The number of digging bouts in a 3-min session was recorded. For this test, there were two outliers (+/−TG 2BC male and +/−TG 2BC female).

#### 2.3.3. Open Field Test

This classic test assesses “emotionality” and is used to measure anxiety-like responses of rodents exposed to stressful environmental stimuli (brightly illuminated open spaces) [74] in addition to overall activity levels. The apparatus used was a white Plexiglas square (50 W × 50 L × 22 H cm), with an open field illuminated to 400 lux in the center. Each animal was placed in the center of the field and several behavioral parameters (distance traveled, velocity, and center time) were recorded during a 60-min observation period and analyzed using Noldus Ethovision XT software. For total distance traveled and average velocity, there were two outliers (+/−TG2BC male and +/−TG2BC female). For time in center, there was one outlier (+/+TG 2BC female).

#### 2.3.4. Forced Swim Test

This classic test is used as a predictive animal model for antidepressant actions of drugs [75,76]. A modification of the test originally described by Porsolt and colleagues [77] and adapted by Lucki [78] was used for our experiments. Mice were individually placed into clear polypropylene cylinders containing 23–25 °C water, 15 cm deep, for 6 min. The number of seconds per minute that each mouse was immobile was recorded. The mouse was considered immobile when no activity was observed other than that required for the mouse to keep its head above the water.

In this test, there were a considerable number of mistrials, defined as a mouse going completely underwater two or more times and exuding bubbles from its nose. To alleviate distress and avoid injury or death, mice were removed from the water the second time this happened and monitored closely until normal behavior returned. This is typically a rare event, and it is unclear why this happened. All groups were affected, but while only 6 2BC were excluded due to mistrials, 25 2BC-CIE mice were excluded due to mistrials.

#### 2.3.5. Tail Flick Test

This test is used to assess pain sensitivity. The mouse was placed in a cylindrical Plexiglas holder with its tail positioned over a window on a platform of the apparatus (Ugo Basile, Varese, Italy). A light beam that provides a radiant heat stimulus is activated, and when the heat becomes painful, the mouse will flick its tail away from the window. The latency to the flick behavior is measured. The mouse was tested 3 times, separated by 1 min for each test, and to prevent tissue damage to the tail, an automatic lamp-cutoff time of 10 s was used.

### 2.4. Protein Assay

#### 2.4.1. Preparation of Protein Samples

After completion of the behavioral testing, the mice were anesthetized with isoflurane, decapitated and the brain removed. The hippocampus was isolated from the remainder of the brain and snap frozen in liquid nitrogen for later processing and protein assays. Protein samples were prepared from the whole hippocampus using standard protocols, as previously described [30]. Briefly, proteins were extracted by sonication in cold lysis buffer containing 50 mM Tris-HCl, pH 7.5, 150 mM NaCl, 2 mM EDTA, 1% Triton X-100, 0.5% NP-40, a Complete Protease Inhibitor Cocktail Tablet (Roche Diagnostics, Mannheim, Germany), and a cocktail of phosphatase inhibitors (Na^+^ pyrophosphate, β-glycerophosphate, NaF, Na^+^ orthovanadate; all from Sigma-Aldrich). After 30-min incubation on ice, the samples were centrifuged at 13,860× *g* for 30 min at 4 °C, and the supernatants collected. Protein concentration in the supernatants was determined using the Bio-Rad Protein Assay Kit (Bio-Rad, Hercules, CA, USA). Aliquots were stored at −80 °C.

#### 2.4.2. Western Blot

Levels of hippocampal proteins were determined using Western blot analysis in a representative number of animals (male and female, randomly selected) from each genotype and treatment group, as described previously [30]. Equal amounts of protein samples were subjected to SDS-PAGE using 4–12% Novex NuPAGE Bis-Tris gels (Invitrogen Life Technologies, Grand Island, NY, USA). Protein samples representative of all genotype and treatment groups were run on each gel. Proteins were transferred to Immobilon-P membranes (Millipore, Billerica, MA, USA), and uniform transfer was assessed using Ponceau S staining (Pierce, Rockford, IL, USA). Membranes were washed and blocked in a 5% casein solution (Pierce), incubated in primary antibody overnight (4 °C), washed, and then incubated (room temperature) in a secondary antibody coupled to horseradish peroxidase (HRP). Protein bands were visualized by chemiluminescence and quantified by densitometry measurements using NIH Image software (NIH Image, version 1.6.3) (http://rsb.info.nih.gov/nih-image/ accessed on 20 January 2023). Membranes were stripped and reprobed for beta-actin. To standardize results, the density of each band was normalized to the density of the band for beta-actin in the same lane of the gel. Normalized data for all samples run on a gel were then normalized to the average normalized value for the control WT hippocampus run on the same gel. Data from different gels were combined according to genotype and treatment and reported as mean ± SEM. 

#### 2.4.3. Antibodies

The following antibodies were used for Western blot studies: a monoclonal antibody produced in rabbits by immunizing them with a fusion protein corresponding to a sequence in the carboxy-terminal of the mouse STAT3 protein (AB#4904; 1:1000; Cell Signaling Technologies, Danvers, MA, USA); a purified polyclonal antibody produced in rabbits by immunizing them with a synthetic phospho-peptide corresponding to the residues surrounding Tyr705 of the mouse STAT3 protein(AB#9131; 1:1000; Cell Signaling Technologies); a purified polyclonal antibody, produced by immunizing rabbits with a synthetic peptide derived from a human glutamic acid decarboxylase 65/67 (GAD65/GAD67; #PA5-38102, 1-2000, Invitrogen, Waltham, MA, USA); a purified mouse monoclonal antibody to the alpha-1 subunit of the gamma-aminobutyric acid (GABA) type A receptor (GABA_A_R), produced by immunizing mice with fusion proteins for amino acids 355–394 of GABA_A_R alpha-1 (375-136, 1-500, UC Davis/NIH NeuroMab Facility); a monoclonal antibody purified to the alpha-5 subunit of GABA_A_R (GABA_A_R alpha-5), produced by immunizing mice with a fusion protein containing a sequence from the cytoplasmic domain of human GABA_A_R alpha-5 subunit (375-401, 1-500, UC Davis/NIH NeuroMab Facility); a monoclonal antibody purified against a fusion protein (amino acids 1-133, cytoplasmic N-terminus) of the mouse vesicular GABA transporter (VGAT)(anti-VGAT clone L118/80, 1-500, UC Davis/NIH NeuroMab Facility); an antibody raised against a fusion protein from the N-terminus of human gephyrin (anti-gephyrin clone L106/83, UC Davis/NIH NeuroMab Facility); a monoclonal antibody purified to subunit 1 of the α-amino-3-hydroxy-5-methyl-4-isoxazolepropionic acid (AMPA) subtype of a glutamate receptor (GluR), produced by immunizing mice with a fusion protein containing a sequence from the extracellular N-terminus region of subunit 1 of rat GluR (GluR1) (75-327, 1-1000, UC Davis/NIH NeuroMab Facility); a purified monoclonal antibody, produced by immunizing mice with a recombinant protein for the full length of the rat postsynaptic density protein 95 (PSD95) (AB#36233, 1-1000, Cell signaling); an affinity isolated antibody to subunit 1 of the N-Methyl-D-Aspartate subtype of glutamate receptor (NMDAR1), produced in a rabbit against a synthetic peptide corresponding to the C-terminal of rat NMDAR1 (amino acids 918-938) (G8913, 1-500, Millipore Sigma); a monoclonal antibody purified to beta-actin, produced by immunizing mice with a synthetic peptide corresponding to a sequences in the amino-terminal of human beta-actin (AB#3700, 1:10,000; Cell Signaling Technology); and a monoclonal antibody purified to beta-actin, produced by immunizing rabbits with a synthetic peptide corresponding to a sequences in the amino-terminal of human beta-actin (AB#4970, 1:5,000; Cell Signaling Technology). Validation for specificity, reproducibility and reliability of the antibodies were carried out by the manufacturer and the user’s lab.

### 2.5. IL-6 and TNF-Alpha Levels

IL-6 and TNF-alpha levels in hippocampal protein samples from TG and WT mice were determined using the DuoSet mouse IL-6 ELISA kit (DY406, R&D Systems, Minneapolis, Minn) or DuoSet mouse TNF-alpha ELISA kit (DY410, R&D Systems), following the manufacturer’s instructions. All samples analyzed by ELISA contained 200 micrograms of protein. IL-6 and TNF-alpha levels were determined according to the respective standard curves, which were run for each ELISA. 

### 2.6. Statistics

Statistical analyses (ANOVA) were conducted using StatView 5.0 (SAS Institute, Inc., Cary, NC, USA). The Tukey–Kramer test was used for post hoc analyses. Statistical significance was set at *p* < 0.05 for all analyses. N = number of animals used. For comparisons showing no treatment effect or a genotype x treatment interaction, data from air and alcohol vapor groups were combined for post hoc tests to provide information about genotype effects. Potential sex differences were assessed for all studies. When significant sex differences were observed, females and males were analyzed separately. Otherwise, data from females and males were combined for analyses. 

## 3. Results

### 3.1. CIE Treatment Escalates 2BC Alcohol Drinking in All Genotypes

The effect of exposure to alcohol vapor (CIE) on free-choice alcohol drinking (2BC) was examined in WT, +/−TG, and +/+TG mice subjected to four cycles of alcohol vapor/abstinence (2BC-CIE). The alcohol drinking levels (2BC) in the WT, +/−TG, and +/+TG mice exposed to air rather than alcohol vapor were used as controls for the effects of CIE on 2BC drinking. The mean (±SEM) BALs (measurements made during each week of vapor treatment) for the treatment period in CIE-treated mice were not significantly different across the genotypes (*F*(2,52) = 0.9, *p* = 0.40); the BALs (mg/dl) were 210 ± 11 (n = 24), 201 ± 7.4 (n = 24), and 185 ± 13 (n = 7) for WT, +/−TG, and +/+TG mice, respectively. 

**Figure 2:**
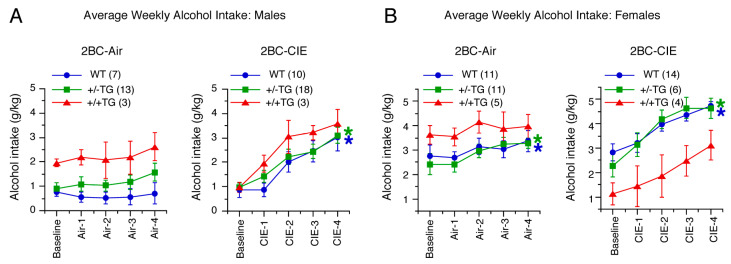
Effects of CIE on 2BC alcohol drinking. (**A**,**B**). Graphs showing effect of cycle on average weekly alcohol intake (mean ± SEM) in male (**A**) and female (**B**) WT, +/−TG, and +/+TG mice in 2BC and 2BC-CIE groups during baseline and four cycles of exposure to air (air 1–4) or CIE (CIE 1–4). * = significant increase in drinking relative to baseline drinking (blue, WT; green, +/−TG; red, +/+TG; data from +/+TG mice were not included in the statistical analyses but are shown for reference). Numbers in parentheses are number of animals studied. In this and all other figures, a statistically significant difference is defined as *p* < 0.05.

A Repeated Measures ANOVA (genotype, treatment, sex, and cycle) showed that females drank significantly more alcohol than males overall (*F*(1,93) = 51.4, *p* < 0.0001) and that there were significant genotype x sex (*F*(2,93) = 4.4, *p* = 0.01) and treatment x sex (*F*(1,93) = 4.2, *p* = 0.04) interactions. Therefore, male and female alcohol drinking were analyzed separately. Two-way ANOVA tests (genotype and treatment) were used to examine baseline drinking levels, and there were no effects of genotype or treatment in males. However, there was an effect of genotype x treatment in females (*F*(2,45) = 3.4, *p* = 0.04), as 2BC-CIE +/+TG females showed lower baseline drinking than 2BC +/+TG females (Figure 2). Because this analysis was performed on baseline drinking before treatment, the lower baseline in the 2BC-CIE +/+TG females was a sampling issue and not an effect of vapor vs. air. This difference was not observed in the overall baseline drinking or in 2BC-CIE males. Indeed, the +/−TG and +/+TG genotypes were determined from a combination of genotype and post-mortem protein analyses, so treatment selection was done before the confirmation of genotype by the protein analyses for the +/−TG and +/+TG mice, leading to an uneven treatment selection in the TG mice. Nevertheless, there were no overall genotype differences in the baseline alcohol intakes. Because of this complication, only results from an analysis of WT and +/−TG females and males are reported below; however, data for +/+TG mice are included in the figure of WT and +/−TF mice (Figure 2).

The alcohol intake from baseline through the four cycles of 2BC-CIE or air were analyzed separately in male and female WT and +/−TG mice using a 3-way Repeated Measures ANOVA (genotype, treatment, and cycle) (Figure 2). In both sexes, there was no effect of genotype (males: *F*(1,44) = 1.6, *p* = 0.22; females: *F*(1,38) = 0.12, *p* = 0.74) but a significant effect of treatment (males: *F*(1,44) = 12.8, *p* = 0.0008; females: *F*(1,38) = 10.6, *p* = 0.002). Significant effects were also observed for cycle (males: *F*(4,176) = 26.4, *p* < 0.0001; females: *F*(4,152) = 21.2, *p* < 0.0001) and cycle x treatment (males: *F*(4,176) = 16.9, *p* < 0.0001; females: *F*(4,152) = 4.8, *p* < 0.001). The other interactions were not significant. 

Because there were significant cycle x treatment interactions, separate ANOVAs were performed within each sex and treatment to examine changes across the CIE/air cycles. There was no effect of cycle on the male 2BC controls (*F*(4,76) = 2.5, *p* = 0.05), whereas there was a significant effect of cycle on the male 2BC-CIE mice (*F*(4,108) = 42.7, *p* < 0.0001). Both the female 2BC control (*F*(4,84) = 4.1, *p* = 0.004) and female 2BC-CIE (*F*(4,76) = 20.4, *p* < 0.0001) mice showed significant effects of cycle. Tukey–Kramer post hoc tests were used to further investigate the cycle effect. The 2BC-CIE males showed significant increases in alcohol intake in cycles 2–4 relative to baseline and cycle 1 and in cycle 4 relative to cycles 2 and 3. In the female 2BC controls, there was a significant increase in drinking in cycle 4 relative to baseline and cycle 1 due to an upward trend in drinking across time even in control mice. However, in the female 2BC-CIE mice, there was a significant increase in alcohol intake in cycles 2–4 relative to baseline and cycle 1. The overall effects of treatment (males: *F*(1,46) = 12.7, *p* = 0.0009; females: *F*(1,40) = 12.5, *p* = 0.001) and treatment x cycle (males: *F*(4,160) = 4.5, *p* = 0.002; females: *F*(4,184) = 16.6, *p* < 0.0001) were significant in both sexes, with alcohol intake being higher in both the male and female 2BC-CIE mice relative to the 2BC mice in cycles 2–4 (Tukey–Kramer tests). Overall, CIE treatment resulted in escalated alcohol intake in males and females of both genotypes (Figure 2).

### 3.2. CIE/Abstinence and Negative Affect/Emotionality

Following the final assessment of the effects of CIE on alcohol drinking, the 2BC and 2BC-CIE WT, +/−TG, and +/+TG mice were subjected to an abstinence period and then behavioral tests were carried out to determine if CIE/abstinence influenced negative affect/emotionality when compared to the respective 2BC group. The testing was started 3 days after the termination of alcohol exposure and included the light/dark transfer, digging, open field, tail flick, and forced swim tests, all of which have been shown to be sensitive to alcohol exposure/abstinence [51,59,60]. The results are summarized in Table 2. Alcohol-naïve mice were not examined in the current studies but have been examined previously [50], and these results are noted for comparison purposes. In the alcohol-naïve mice, data from males and females were combined as sex did not impact the results. 

#### 3.2.1. Light/Dark Transfer Test

**Figure 3:**
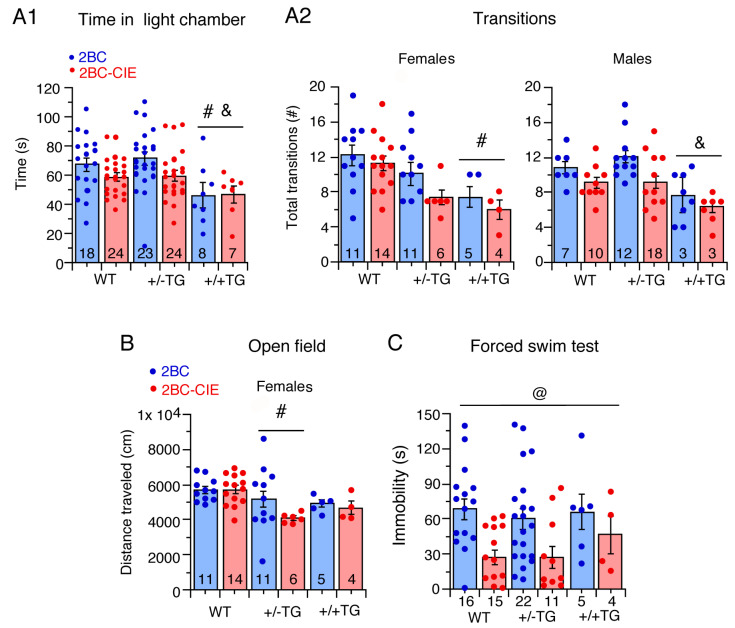
Graphs showing effects of 2BC and 2BC-CIE on behavioral tests for negative affect/emotionality during the abstinence period. CIE actions are reflected in differences between effects of 2BC and 2BC-CIE. Behavioral tests include light/dark transfer (measures of time in light chamber) (**A1**), and total number of transitions between dark and light chamber (**A2**), open field test (distance traveled) (**B**) and immobility time (forced swim test) (**C**). In this and other figures, bar graphs show mean ± SEM values and individual data points. Numbers within bars or under bars are number of animals tested. Bars underneath symbols for significance indicate 2BC plus 2BC-CIE data are combined (i.e., no treatment effect). # = significantly different from WT, & = significantly different from +/−TG, @ = overall 2BC-CIE significantly lower than 2BC.

There was a significant effect of genotype *F*(2,92) = 4.8, *p* = 0.01) on the time spent in the light chamber but no effect of treatment (i.e., 2BC-CIE vs. 2BC) or sex and no interactions. The Tukey–Kramer post hoc testing showed that the alcohol-exposed/abstinent +/+TG mice spent less time in the light chamber relative to the alcohol-exposed/abstinent +/−TG and WT mice, indicative of greater emotionality/anxiety-like behavior in the +/+TG mice (Figure 3(A1)). This effect may reflect an action of alcohol exposure/abstinence on emotionally/anxiety-like behavior in the +/+TG mice, as in our previous studies of alcohol-naïve mice there were no significant differences in time spent in the light chamber between +/+TG and +/−TG or WT mice [50]. 

There was a significant effect of genotype (*F*(2,92) = 9.7, *p* = 0.0002) and treatment (*F*(1,92) = 7.3, *p* = 0.008) on dark-to-light compartment transitions and a genotype x sex interaction (*F*(2,92) = 3.8, *p* = 0.02) but no genotype x treatment interaction. Tukey–Kramer post hoc testing for genotype showed that +/+TG mice made fewer dark-to-light transitions relative to +/−TG and WT mice, again indicative of greater emotionality/anxiety-like behavior in the +/+TG mice. Further examination of the genotype x sex interaction revealed significant effects of genotype on both females (*F*(2,48) = 7.55, *p* = 0.001) and males (*F*(2,50) = 3.89, *p* = 0.03). The female +/+TG mice made fewer transitions than the WT mice, and the male +/+TG mice made fewer transitions than the +/−TG mice (Figure 3(A2)). In the alcohol-naïve mice examined in our previous study [50], there was no effect of sex, therefore males and females were not studied separately. In those studies, +/+TG mice showed greater emotionality/anxiety-like behavior than WT mice.

#### 3.2.2. Digging Test

There were no significant effects of genotype, sex, treatment, or any interaction between these on the time spent digging or the number of digging bouts (not shown). This was unexpected, as abstinence from alcohol is often associated with increased digging behavior [55,79,80]. In our previous study of alcohol-naïve mice [50], +/+TG mice showed fewer digging bouts than WT mice, suggesting the possibility that alcohol blocked/reduced this effect.

#### 3.2.3. Open Field Test

There was a significant effect of genotype on distance traveled (*F*(2,93) = 3.1, *p* = 0.04) and a significant genotype × sex interaction *F*(2,93) = 3.4, *p* = 0.03). Despite the significant effect of genotype in the overall ANOVA, there were no individual genotype differences in post hoc analyses. The genotype x sex interaction follow-up analyses revealed a significant effect of genotype in females (*F*(2,48) = 4.29, *p* = 0.02), with +/−TG moving less than WT mice, but no effect of genotype in males. These results indicate that alcohol exposure/abstinence induces greater anxiety-like behavior/emotionality in female +/−TG mice than in female WT mice.

There was an overall sex difference in velocity (*F*(1,93) = 4.3, *p* = 0.04), with females moving faster than males, but no significant effects in center time. In our previous study of alcohol-naïve mice, +/+TG mice showed lower velocity than WT mice but no difference in center time [50]. 

#### 3.2.4. Forced Swim Test

The results obtained from the mice that successfully completed the test showed a significant effect only for treatment (*F*(1,62) = 7.7, *p* = 0.007), with 2BC-CIE mice having decreased immobility times relative to 2BC mice (Figure 3C). Increased climbing and struggling could have been a factor in these mice. In our previous study of alcohol-naïve mice, +/+TG mice showed greater immobility compared to WT mice [50].

#### 3.2.5. Tail Flick Test

An average of the three trials was used for the statistical analysis, and the only significant effect was found for the treatment group(*F*(1,93) = 4.4, *p* = 0.04). There were no significant effects of genotype, sex, or any interaction between these or treatment in the tail flick test (not shown). Alcohol-naïve mice were not tested for tail flick in our previous study.

Taken together, the results from these behavioral studies show that when measured during the abstinence period, there were overall effects of the treatment on light dark transfer transitions, forced swim immobility, and tail flick latency, indicative of an effect of alcohol abstinence on negative affect/emotionality. However, regarding genotype, the behavior of the 2BC-CIE-treated mice was not significantly different from that of the 2BC mice. Since all mice in this study had alcohol experience, the genotypic differences that were observed likely represent an increase in negative affect/emotionality in the transgenic mice associated with alcohol exposure/abstinence (Table 2). 

### 3.3. Effects of 2BC and 2BC-CIE on Neuroimmune Factors in the Hippocampus

Our previous studies showed that CIE/withdrawal (i.e., no 2BC) altered the levels of IL-6, IL-6 signal transduction partners, and several synaptic proteins compared to alcohol-naïve control mice in the hippocampus and cerebellum when measurements were made 24 h after termination of CIE [27,30,81]. In the current studies, animals were sacrificed nine days after the cessation of 2BC and 2BC-CIE exposure. To determine if neurochemical changes were evident after this alcohol exposure paradigm and a more prolonged abstinence period, the levels of several neurochemicals previously shown to be altered by CIE were examined. The results are summarized in Table 3, along with results from our previous studies of CIE-treated hippocampi and cerebellum for comparison purposes. 

#### 3.3.1. IL-6 Levels

**Figure 4:**
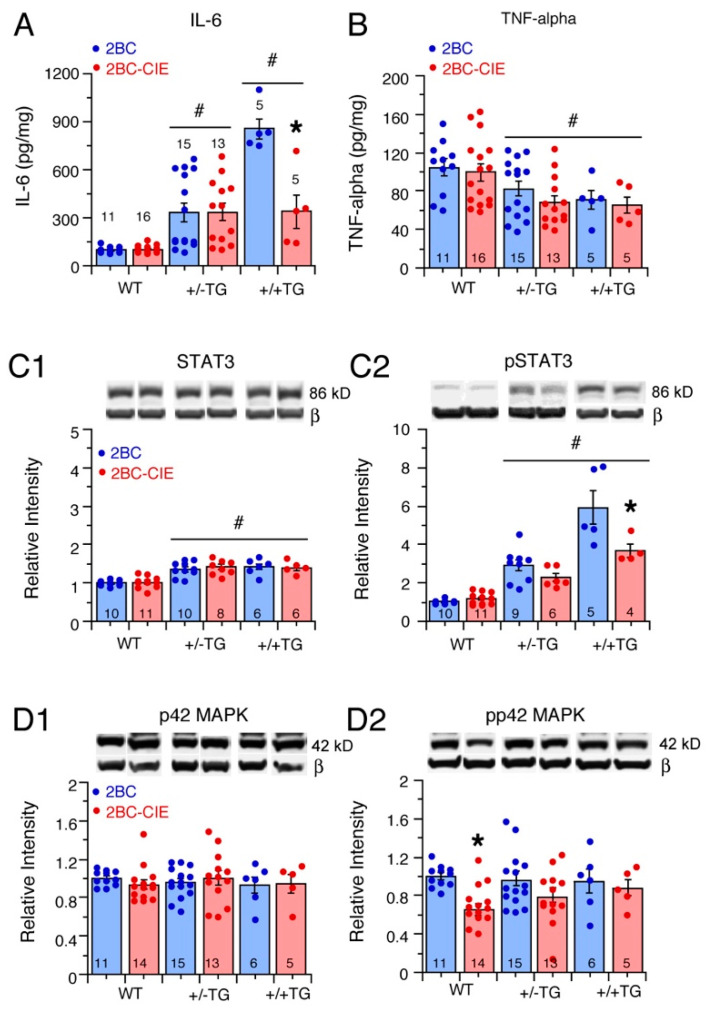
Effects of 2BC and 2BC-CIE on neuroimmune-related proteins. (**A**,**B**). Mean (± SEM) values for levels of IL-6 (**A**) and TNF-alpha (**B**) measured by ELISA in hippocampi from 2BC- and 2BC-CIE-treated WT, +/−TG, and +/+TG mice. (**C**,**D**). Mean (± SEM) levels of IL-6 signal transduction partners STAT3 (**C1**), pSTAT3 (**C2**), p42MAPK (**D1**), and pp42MAPK (**D2**), measured by Western blot analysis in hippocampi from 2BC- and 2BC-CIE-treated WT, +/−TG, and +/+TG mice. Representative Western blots are inserted above the corresponding data bars. Β = beta-actin (42 kD). The source Western blots are included in the Supplemental section (Figure S1). # = significantly different from WT. * = significant difference between 2BC and 2BC-CIE of the same genotype. Note, on these and other graphs showing data points, some data points may be masked by overlapping values. Numbers within or above the bars show the number of animals in the sample.

The IL-6 levels in the hippocampi from the WT, +/−TG, and +/+TG mice showed a significant genotype (*F*(2,61) = 25.9, *p* < 0.0001) and treatment (*F*(1,61) = 6.0, *p* = 0.02) effect and genotype x treatment interaction (*F*(2,61) = 6.2, *p* = 0.03). A post hoc analysis of the 2BC groups showed significant genotype differences in IL-6 levels that reflected the IL-6 gene–dose relationship (i.e., all three genotypes significantly different from each other), with +/+TG mice showing the highest levels of IL-6 and WT showing the lowest (Figure 4A). The IL-6 levels in the 2BC-CIE group were also higher in the +/−TG and +/+TG mice than in the WT mice, but the levels in the +/−TG and +/+TG mice were not significantly different. The IL-6 levels in the 2BC-CIE +/+TG group were significantly lower than in the 2BC +/+TG group, indicating that CIE treatment reduced IL-6 levels in the +/+TG group (Figure 4A). 

#### 3.3.2. TNF-Alpha Levels

The hippocampal levels of TNF-alpha in the WT, +/−TG, and +/+TG mice subjected to 2-BC or 2BC-CIE treatment showed a significant genotype effect (*F*(2,61) = 7.3, *p* = 0.001) but no treatment effect (*F*(1,61) = 1.5, *p* = 0.23) or genotype x treatment interaction (*F*(2,61) = 0.13, *p* = 0.88). A post hoc analyses showed that TNF-alpha levels were significantly lower in the +/−TG and +/+TG mice compared to the WT mice (Figure 4B).

### 3.4. Effects of 2BC and 2BC-CIE on IL-6 Signal Transduction Partners STAT3 and p42/44MAPK in the Hippocampus

The biological effects of IL-6 are transduced primarily by two signaling pathways that couple to IL-6 receptors (IL-6Rs) through JAK, one centered on STAT3 and the other centered on p42/44 MAPK. The involvement of these pathways in the effects of the alcohol/abstinence treatment paradigm was assessed by a comparison of the levels of the total (STAT3; p42/44 MAPK) and activated forms of these proteins (pSTAT3; pp42/44 MAPK) in the hippocampus of 2BC and 2BC-CIE WT, +/−TG, and +/+TG mice. The results are summarized in Table 3.

**STAT3**. The STAT3 levels showed a significant genotypic difference (*F*(2,44) = 35.3, *p* < 0.0001) but no treatment effect (*F*(1,44) = 0.11, *p* = 0.74), or genotype x treatment interaction (*F*(2,44) = 0.42, *p* = 0.66). A post hoc analysis showed that the STAT3 levels were significantly higher in the +/−TG and +/+TG hippocampi compared to the STAT3 levels in the WT hippocampus (Figure 4(C1)).

**pSTAT3.** The effects on hippocampal pSTAT3 levels were reflective of the effects on IL-6 levels (Figure 4A), with a significant genotype difference (*F*(2,39) = 89.1, *p* < 0.0001), treatment effect (*F*(1,39) = 12.5, *p* = 0.001), and genotype x treatment interaction (*F*(2,39) = 7.1, *p* = 0.002) (Figure 4(C2)). A post hoc analysis showed significant genotype differences for both the 2BC and 2BC-CIE groups, with the hippocampus of the +/+TG mice showing the highest levels of pSTAT3 and WT mice showing the lowest. Regarding genotypes, there was no significant difference in the pSTAT3 levels between the 2BC and 2BC-CIE groups for the WT and +/−TG hippocampi, but in the +/+TG mice, the pSTAT3 levels were significant lower in the 2BC-CIE group compared to the 2BC group (Figure 4(C2)), which is consistent with the lower IL-6 levels in the +/+TG mice (Figure 4A).

**p42 MAPK.** The hippocampal levels of p42 MAPK showed no significant genotypic difference (*F*(2,58) = 0.16, *p* = 0.86), treatment effect (*F*(1,58) = 0.0, *p* = 0.99), or genotype x treatment interaction (*F*(2,58) = 0.58, *p* = 0.56) (Figure 4D1).

**pp42 MAPK.** The hippocampal levels of pp42 MAPK showed no significant genotypic difference (*F*(2,58) = 0.22, *p* = 0.81) but a significant treatment effect (*F*(1,58) = 7.5, *p* = 0.008) and no genotype x treatment interaction (*F*(2,58) = 0.99, *p* = 0.38). In a post hoc analyses, the WT pp42 levels were significantly lower in the 2BC-CIE group compared to the 2BC group (Figure 4D1).

**p44 MAPK.** The hippocampal levels of p44 MAPK showed a significant genotypic difference (*F*(2,58) = 4.2, *p* = 0.02) but no treatment effect (*F*(1,58) = 0.8, *p* = 0.99) or genotype x treatment interaction (*F*(2,58) = 0.03 *p* = 0.97). A post hoc analysis showed that the p44 MAPK levels in hippocampi from the +/+TG mice were significantly lower than in +/−TG mice (not shown).

**pp44 MAPK.** The hippocampal levels of pp44 MAPK showed no significant genotype effect (*F*(2,58) = 0.99, *p* = 0.38), significant treatment effect (*F*(1,58) = 0.8, *p* = 0.37), or genotype x treatment interaction (*F*(2,58) = 0.39 *p* = 0.68) (not shown). 

### 3.5. Effects of 2BC and 2BC-CIE on Inhibitory and Excitatory Synaptic Proteins in the Hippocampus

Inhibitory GABA-mediated and excitatory glutamate-mediated synaptic transmissions are known to be key sites of alcohol action in the brain. A variety of molecular components that are essential for synaptic transmission are affected by alcohol, including transmitter receptor subunits, transmitter transporters, synthetic enzymes for transmitters, and structural components, with the net effect resulting in an alcohol-induced enhancement or depression of synaptic transmission and/or synaptic plasticity depending on the alcohol level, route, and duration of administration, the brain region, and other factors. Several of these synaptic components were examined to determine if the levels were altered by 2BC or 2BC-CIE in a genotypic manner. The results are summarized in Table 4, along with results from our previous studies for comparison purposes.

For inhibitory synaptic transmission, the proteins examined included GABA_A_R subunits alpha-1 and alpha-5, which are components of GABA_A_Rs that mediate phasic (alpha-1) or tonic (alpha-5) inhibitory synaptic transmission; VGAT, the transporter that imports GABA into synaptic vesicles; GAD67/65, two enzymes that are expressed in inhibitory neurons and are involved in maintaining levels of GABA by catalyzing the conversion of glutamate to GABA; and gephyrin, the main postsynaptic scaffolding protein at inhibitory synapses, where it plays a key role in regulating the distribution and clustering of GABA_A_Rs. 

For excitatory synaptic transmission, the proteins examined included GluR1, a subunit of the AMPA receptor; NR1, a subunit of the NMDA receptor, both of which are components of the receptors that mediate excitatory synaptic transmission; and PSD95, a scaffolding protein in the postsynaptic density at excitatory synapses. The results are summarized in Table 3.

#### 3.5.1. Inhibitory Synaptic Proteins

**Figure 5:**
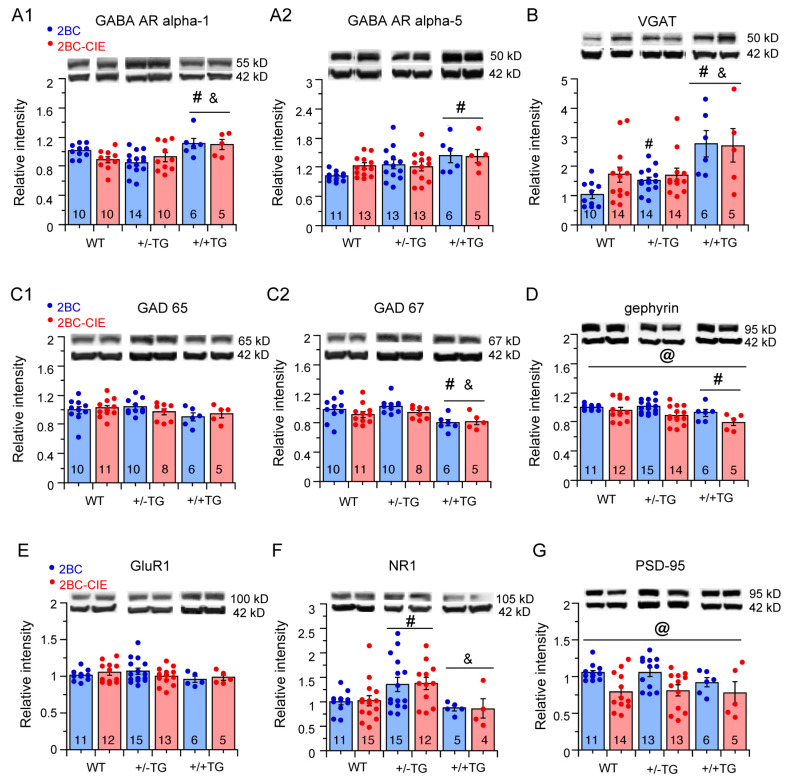
Effects of 2BC and 2BC-CIE on synaptic proteins. (**A**–**G**). Mean (± SEM) values for levels of GABA_A_R alpha-1 (**A1**) and alpha-5 (**A2**), VGAT (**B**), GAD65 (**C1**) and GAD 67 (**C2**), gephyrin (**D**), GluR1 naïve, NR1 (**F**), and PSD-95 (**G**) determined by Western blot in WT, +/−TG, and +/+TG mice exposed to 2BC/abstinence or 2BC-CIE-abstinence protocols. The source Western blots are included in the Supplemental section (Figure S2). # = significantly different from WT, & = significantly different from +/−TG, @ = overall 2BC-CIE significantly lower than 2BC.

**GABA_A_R alpha-1**. The hippocampal levels of GABA_A_R alpha-1 showed a significant genotype difference (*F*(2,49) = 7.2, *p* = 0.002) but no significant treatment effect (*F*(1,49) = 0.25, *p* = 0.62) or genotype x treatment interaction (*F*(2,49) = 2.1, *p* = 0.13). In post hoc tests, hippocampal GABA_A_R alpha-1 levels were significantly higher in hippocampi from +/+TG mice than in hippocampi from either +/−TG or WT mice (Figure 5(A1)).

**GABA_A_R alpha-5.** The hippocampal levels of GABA_A_R alpha-5 showed a significant genotypic difference (*F*(2,55) = 4.3, *p* = 0.02) but no significant treatment effect (*F*(1,55) = 0.61, *p* = 0.44) or genotype x treatment interaction (*F*(2,55) = 0.63, *p* = 0.53). In post hoc tests, GABA_A_R alpha-5 levels in 2BC +/+TG mice were significantly higher than in 2BC WT mice (Figure 5(A2)). 

**Vesicular transporter for GABA (VGAT)**. The hippocampal levels of VGAT, which is highly concentrated on the synaptic vesicles of GABAergic neurons, showed a significant genotypic difference (*F*(2,57) = 12.3, *p* < 0.0001) but no significant treatment effect (*F*(1,57) = 0.7, *p* = 0.42) or genotype x treatment interaction (*F*(2,57) = 1.1, *p* = 0.36). In post hoc tests, VGAT levels in +/+TG mice were significantly higher than in hippocampi from +/−TG or WT mice (Figure 5B).

**GAD65/67.** The hippocampal levels of GAD65 showed no significant genotypic difference (*F*(2,44) = 1.7, *p* = 0.18), treatment effect (*F*(1,44) = 0.08, *p* = 0.78), or genotype x treatment interaction (*F*(2,44) = 0.5, *p* = 0.62) (Figure 5(C1)). For hippocampal levels of GAD67, there was a significant genotypic difference (*F*(2,44) = 7.2, *p* = 0.002), but no significant treatment effect (*F*(1,44) = 1.6 *p* = 0.15) or genotype x treatment interaction (*F*(2,44) = 0.22, *p* = 0.80). A post hoc analysis showed a significantly lower level of GAD67 in hippocampi from +/+TG mice compared to hippocampi from +/−TG or WT mice.

**Gephyrin.** There was a significant genotypic difference (*F*(2,57 = 4.8, *p* = 0.01) and treatment effect (*F*(1,57) = 9.8, *p* = 0.003) for the levels of hippocampal gephyrin but no significant genotype x treatment interaction (*F*(2,57) = 1.1, *p* = 0.34). A post hoc analysis showed that the gephyrin levels in hippocampi from +/+TG mice were significantly lower than from WT mice. In addition, the overall gephyrin levels were significantly lower in 2BC-CIE/abstinence-treated mice that 2BC/abstinence-treated mice (Figure 5D).

#### 3.5.2. Excitatory Synaptic Proteins

**GluR1.** There was no significant genotypic difference (*F*(2,55) = 0.5, *p* = 0.60), treatment effect (*F*(1,55) = 0.3, *p* = 0.58) or genotype x treatment interaction (*F*(2,55) = 1.7, *p* = 0.19) for the levels of hippocampal GluR1 (Figure 5E).

**NR1.** There was a significant genotypic difference (*F*(2,56) = 7.2, *p* = 0.02) but no significant treatment effect (*F*(1,57) = 0.1, *p* = 0.75) or genotype x treatment interaction (*F*(2,57) = 0.07, *p* = 0.93) for the hippocampal levels of NR1. A post hoc analysis showed that levels of NR1 were significantly higher in the +/−TG mice than either the +/+TG or WT mice (Figure 5F).

**PSD-95.** There was no significant genotypic difference (*F*(2,56) = 0.8, *p* = 0.46) or genotype x treatment interaction (*F*(2,56) = 0.02, *p* = 0.98) for hippocampal levels of PSD-95, but there was a significant treatment effect (*F*(1,52) = 11.8, *p* = 0.001). Overall, PSD95 levels were lower in hippocampi from the 2BC-CIE mice compared to 2BC mice (Figure 5G).

## 4. Discussion

The current studies examined the impact of neuroimmune status with respect to IL-6 on 2BC alcohol drinking, the consequences of CIE on 2BC alcohol drinking, and the behavioral and neurochemical effects of 2BC and 2BC-CIE during the abstinence period. The mice subjected to the 2BC drinking protocol alone are considered non-dependent [67], whereas the 2BC-CIE protocol has been shown to induce changes in mice associated with alcohol dependence such as increase in alcohol drinking and altered behaviors. The studies were carried out in WT, +/−TG, and +/+TG mice. The TG mice express elevated levels of IL-6 in the brain in a gene dose manner as a consequence of targeted genetic manipulation of IL-6 expression in astrocytes. These mice were used as a model for neuroimmune status of the brain with respect to IL-6 in subjects who persistently consume high levels of alcohol, which is known to increase IL-6 levels in the brain. Our previous studies of alcohol-naïve WT, +/−TG, and +/+TG mice showed neurochemical, neuroimmune, and neurophysiological differences between these genotypes that were altered by acute alcohol and/or CIE, indicative of a role for alcohol–neuroimmune interactions with respect to IL-6 in the actions of alcohol [27,30,57]. In the current studies, several behavioral and neurochemical differences were observed between the WT, +/−TG, and +/+TG mice subjected to alcohol exposure/abstinence. These results are consistent with results from our previous studies using other alcohol exposure paradigms and support the idea that IL-6–alcohol interactions contribute to the behavioral and neurochemical effects of alcohol exposure/abstinence.

### 4.1. Alcohol Drinking

Female mice are known to consume more alcohol than males (e.g., [82,83,84]), as was the case for both the WT and TG mice in the current studies. Both females and males from all genotypes within the 2BC-CIE group showed escalated 2BC alcohol consumption compared to baseline 2BC consumption within the same genotype (WT, +/−TG, or +/+TG). Thus, all genotypes responded as expected for animals that developed dependence, suggesting that neuroimmune status with respect to IL-6 does not play a role in escalading alcohol consumption, at least under the conditions of our 2BC-CIE study. However, within the 2BC group, a small but significant escalation in alcohol consumption was observed in the female 2BC +/−TG mice compared to the baseline 2BC +/−TG consumption, an effect not observed for female 2BC WT or +/+TG mice. This result may reflect a greater susceptibility to a binge pattern of alcohol consumption in the +/−TG mice relative to either WT or +/+TG mice, and perhaps an early stage in the development of alcohol dependence. 

### 4.2. Tests for Negative Affect/Emotionality

The results from the behavioral studies showed that when measured during the abstinence period, there were several signs of increased negative affect/emotionality in the 2BC-CIE mice relative to 2BC mice. However, within genotypes, these effects were not significant. The lack of behavioral significance between the 2BC-CIE- and 2BC-treated mice of the same genotype may reflect the difficulty in demonstrating the effects of alcohol on negative affective symptoms in mice during abstinence [51,55,60,85]. However, genotypic and sex differences were observed for some behaviors that were not observed in our previous behavioral studies of alcohol-naïve mice, consistent with a role for neuroimmune status with respect to IL-6 in the behavioral effects of alcohol/abstinence.

In the studies of alcohol-naïve mice, the behavior of +/−TG mice was not significantly different from that of WT mice in a variety of tests for negative affect/emotionality [50]. Similar results were observed in the current study for the behaviors of alcohol-treated/abstinent +/−TG vs. alcohol-treated/abstinent WT mice, with the exception that alcohol-treated/abstinent female WT mice moved significantly farther than alcohol-treated/abstinent female +/−TG mice in the open field test, indicative of greater anxiety-like behavior/emotionality in the alcohol-treated/abstinent female +/−TG mice. 

In contrast, the alcohol-naïve +/+TG mice showed several behavioral differences when compared to the alcohol-naïve WT mice, and these differences were not observed for alcohol-treated/abstinent +/+TG mice, with the exception of a lower number of transitions in the light/dark transfer test, which was observed for both alcohol-naïve and alcohol-treated/abstinent +/+TG mice when compared to the WT mice of the same treatment group (Table 1). In addition, there was no significant difference in the time spent in the light compartment between alcohol-naïve +/+TG and alcohol-naïve WT or +/−TG mice, whereas time spent in the light compartment was significantly lower in alcohol-treated/abstinent +/+TG mice compared to alcohol-treated/abstinent WT or +/−TG mice, indicative of greater anxiety-like behavior/emotionality in the alcohol-treated/abstinent +/+TG mice. 

These results are consistent with the impact of neuroimmune status with respect to IL-6 in the effects of alcohol/abstinence on anxiety-like behavior/emotionality in the +/−TG and +/+TG mice. However, as alcohol-naïve mice were not included in the current studies, the difference between alcohol-naïve and alcohol-treated/abstinent mice will need to be confirmed in studies that include both treatment groups. 

### 4.3. Neuroimmune Factors

The +/−TG and +/+TG mice showed a significant elevation in the hippocampal levels of the IL-6 and IL-6 signal transduction partner pSTAT3 compared to the WT mice of the same treatment group (2BC/abstinent or 2BC-CIE/abstinent). However, a genotype effect of alcohol exposure/abstinence was only observed for the +/+TG group, where both IL-6 and pSTAT3 levels were significantly reduced by the 2BC-CIE treatment compared to the 2BC controls. As IL-6 signal transduction involves the activation of pSTAT3, this result is consistent with the CIE/abstinence reduction in IL-6 levels contributing to the reduction in pSTAT3 levels. This result also identifies a genotypic difference in the sensitivity of +/−TG vs. +/+TG mice to CIE/abstinence, as the +/−TG mice did not show a difference in the effects of 2BC/abstinence and 2BC-CIE/abstinence on IL-6 and pSTAT3 levels. However, in our previous study of CIE/24 h abstinent +/−TG mice (no 2BC), pSTAT3 levels were reduce in the hippocampus of +/−TG mice relative to alcohol-naïve +/−TG mice, whereas in WT mice, hippocampal pSTAT3 levels were increased [30] (Table 3).

In contrast, the pp42 levels, which reflect another leg of the IL-6 signal transduction pathway (i.e., in contrast to the STAT3 leg), were lower in hippocampi from 2BC-CIE WT mice compared to levels in hippocampi from 2BC WT mice. This effect of CIE on WT mice is consistent with our previous studies of the CIE effects on the hippocampus of rats that also showed a decrease in pp42 levels with CIE treatment [86] and for the +/−TG mice, a decrease in IL-6, pSTAT3, and pp42 MAPK levels in the cerebellum with CIE treatment [27] compared to levels in the same genotype and brain region in alcohol-naïve mice (Table 3). 

pSTAT3 is expressed by both neurons and the glia of the brain, is utilized by a variety of signaling factors, and is known to play an important role in brain function and health [73,87,88,89]. Therefore, the effects of alcohol exposure/abstinence on pSTAT3 levels could significantly impact the effects of alcohol on a variety of targets. Outside of our studies, few studies have examined the effects of alcohol on pSTAT3 levels in the brain. Interestingly, a recent report using a transcriptome approach showed that pSTAT3 levels were increased in the hippocampus of rats during withdrawal from chronic alcohol (9% ethanol liquid diet for ~15 days) [29]. The increase in pSTAT3 in the hippocampus primarily occurred in the astrocytes. In behavioral studies, the authors showed that a STAT3 antagonist blocked both the chronic alcohol activation of STAT3 and sucrose preference, which was used as a behavioral measure of anhedonia, a cluster of well-known negative affect behavioral symptoms associated with alcohol withdrawal, thereby linking STAT3 with the behavioral effects of alcohol [29]. Recent studies have also linked increased levels of proinflammatory cytokines, including IL-6, during withdrawal from chronic alcohol (14 days of ethanol by gavage, 19 days of withdrawal) as important factors in depressive-like behaviors associated with alcohol abstinence [90]. 

In contrast to the alcohol exposure/abstinence-induced increase in IL-6 levels, TNF-alpha levels, which are thought to play a role in depressive-like behaviors [91], were reduced in alcohol-exposed/abstinent +/−TG and +/+TG mice compared to hippocampal levels in alcohol-exposed/abstinent WT mice. The effects of alcohol-exposure/abstinence on TNF-alpha levels in WT mice could not be determined in the current study as alcohol-naïve mice were not included for comparison. This effect of alcohol exposure/abstinence may contribute to the reduced immobility (forced swim test) in the 2BC-CIE-treated mice compared to the 2BC mice, which is indicative of reduced depressive-like behavior. Recent studies showed that withdrawal from chronic alcohol increased depressive-like behaviors (forced swim test and tail suspension test) in mice, an effect associated with an increase in the brain levels of TNF-α, IL-1β, and IL-6 [90]. Blocking the increased expression of the proinflammatory cytokines also blocked the depressive-like behavior [90]. 

Thus, CIE/abstinence, 2BC-CIE/abstinence, and 2BC/abstinence can all impact levels of IL-6, TNF-alpha, pSTAT3, and pp42 MAPK, indicating that they are targets of alcohol actions and/or IL-6–alcohol interactions and supporting the idea that alcohol and the neuroimmune status of the brain have complex interactions that can affect both neuroimmune status and actions of alcohol.

### 4.4. Synaptic Proteins

In the alcohol-exposed mice, there was no significant difference in the levels of synaptic proteins between 2BC/abstinent and 2BC-CIE/abstinent mice, with the exception that the overall PDS95 and gephyrin levels were lower in the hippocampus of 2BC-CIE/abstinent mice than in the hippocampus of 2BC/abstinent mice, indicative of a sensitivity of these two synaptic proteins to alcohol dose. However, genotypic differences were observed, indicative of interactions between alcohol/abstinence and neuroimmune status with respect to IL-6. The synaptic proteins measured in these studies are necessary to synaptic transmission in the brain and, consequently, behavior. Thus, the alcohol/abstinence-induced changes in the levels of the expression of these proteins could contribute to the behavioral effects of 2BC and/or 2BC-CIE observed in these studies. 

The GABA_A_R-α1 levels were higher in the hippocampus of alcohol-exposed/abstinent +/+TG mice than in the hippocampus of alcohol-exposed/abstinent WT or +/−TG mice, and the NR1 levels were significantly higher in the hippocampus of alcohol-exposed/abstinent +/−TG mice compared to alcohol-exposed/abstinent +/+TG or WT mice. These differences were not observed between alcohol-naïve +/−TG and +/+TG mice in our previous study [50] or, for GABA_A_R-α1 levels, in the hippocampus of +/−TG and WT mice exposed to CIE/24 h abstinence compared to alcohol-naïve mice of the same genotype, or for GABA_A_R-α1 levels between alcohol-naïve +/−TG and alcohol-naïve WT mice [30]. Thus, these genotypic differences could reflect actions of the alcohol exposure/abstinence protocol used in the current studies.

In contrast to GABA_A_R-α1, hippocampal GABA_A_R-α5 levels were higher in the hippocampus of alcohol-exposed/abstinent +/+TG mice than in alcohol-exposed/abstinent WT mice, a relationship similar to that observed for the hippocampus of alcohol-naïve mice (Table 1). GAD67, VGAT, and gephyrin levels in the hippocampus of alcohol-exposed/abstinence +/−TG or +/+TG mice also showed genotypic relationships similar to that observed for the respective alcohol-naïve mice. Therefore, for these proteins, it is unknown if the genotypic relationships observed in the alcohol-exposed/abstinent mice represent a treatment effect or the relationship in alcohol-naïve mice (i.e., no treatment effect). Studies involving cohorts of both alcohol-naïve and alcohol-exposed mice are necessary to resolve this issue, as both WT and TG mice in the current study were alcohol-treated. 

In contrast to +/+TG mice, GABA_A_R-α5 levels in alcohol-exposed/abstinent +/−TG were not significantly different from alcohol-exposed/abstinent WT mice, whereas in the hippocampus of CIE/24 hr abstinent mice, GABA_A_R-α5 levels were reduced in both the +/−TG and WT mice compared to their respective alcohol-naïve controls, and in alcohol-naïve mice, GABA_A_R-α5 levels were higher than in alcohol-naïve WT mice. 

Taken together, the alcohol/abstinence-induced differences in the genotypic relationships for hippocampal GABA_A_R-α1, GABA_A_R-α5, and NR1 levels in WT, +/−TG, and +/+TG mice are indicative of alcohol/abstinence interactions with neuroimmune status. For the GABA_A_Rs, these interactions could result in a shift in the relative levels of tonic (mediated by GABA_A_R α-5-containing receptors) vs. phasic (mediated by GABA_A_R α-1-containing receptors) GABAergic inhibition in the hippocampus [92,93] and potentially contribute to the behavioral changes observed in the current studies.

Previous studies have shown the effects of chronic alcohol exposure on GABA_A_R expression with results varying depending on brain region and exposure paradigms [24,94,95,96]. These differences are thought to reflect different mechanisms of regulation [95], which could apply to the different actions of alcohol/abstinence observed in our studies. 

## 5. Conclusions

Overall, the results from the current study demonstrates that interactions between alcohol and the neuroimmune status of the brain impact the actions of alcohol on brain neurochemistry and behavior. These results complement our previous studies by providing information on the effects of a different alcohol exposure protocol on some of the same endpoints previously studied. The results extend the current understanding of the role of neuroimmune–alcohol interactions in the effects of different alcohol exposure paradigms on the brain. The information also contributes to the understanding of the consequences of elevated levels of IL-6 in the brain, which occurs in a variety of conditions that negatively affect brain function and are often co-morbid with AUD, such as depressive-like behaviors. 

## Figures and Tables

**Table 1:** Timing of behavioral testing.

Behavioral Test	Post Alcohol Day of Testing	Method of Measuring Behavior	Used to Assess
light/dark transfer test	3 days post alcohol	scored by experimenter blind to genotype and treatment	anxiety-like behavior, exploratory drive
digging test	4 days post alcohol	live scored by experimenter blind to genotype and treatment	response to novelty and possibly irritability
open field test	5 days post alcohol	Noldus Ethovision XT v14 software	emotionality, activity
forced swim test	6 days post alcohol	scored by experimenter blind to genotype and treatment	helpless-like behavior
tail flick test	7 days post alcohol	automated scoring by device (Ugo Basile)	pain sensitivity

**Table 2:** Summary of results from behavioral studies.

Behavioral Test (Mice 3–8 Days Withdrawn)	TG+/− vs. WT			TG +/+ vs. WT			TG +/+ vs. TG+/−			2BC-CIE vs. 2BC		
	Naïve ^1^	2BC	2BC- CIE	Naïve ^1^	2BC	2BC- CIE	Naïve ^1^	2BC	2BC- CIE	WT	+/− TG	+/+ TG
1. Light/dark transfer												
-time spent in light	ns	overall ns		ns	overall **↓**		ns	overall **↓**		overall ns		
-#of transitions	ns	overall ns		**↓**	M & F **↓**		ns	overall M **↓**		overall M **↓**		
2. Open field test												
-distance	ns	overall F **↓**		**↓**	overall ns		ns	overall ns		overall ns		
-velocity	ns	overall ns		**↓**	overall ns		ns	overall ns		overall ns		
-center time	ns	overall ns		ns	overall ns		ns	overall ns		overall ns		
3. Digging bouts	ns	overall ns		**↓**	overall ns		ns	overall ns		overall ns		
4. Forced swim test												
-Immobility	ns	overall ns		**↑**	overall ns		ns	overall ns		overall **↓**		
5. Tail flick test	ns	overall ns		ns	overall ns		ns	overall ns		overall **↓**		

**↓** = significantly lower, **↑** = significantly higher, ns—no significant difference; overall is used when there were no treatment effects. ^1^ data from [50].

**Table 3:** Summary of alcohol-induced changes in mean levels of hippocampal or cerebellar proteins.

Protein	Alcohol-Naïve ^1^ Hippocampus		Alcohol-Exposed, 3–8 Days Abstinent Hippocampus							CIE (24 Withdrawn)			
	TG vs. WT		2BC TG vs. WT		2BC-CIE TG vs. WT		2BC-CIE vs. 2BC			Hippocampus ^2^		Cerebellum ^3^	
	+/− TG	+/+ TG	+/− TG	+/+ TG	+/− TG	+/+ TG	WT	+/− TG	+/+ TG	WT vs. Naïve	+/−TG vs. Naïve	WT vs. Naïve	+/−TG vs. Naïve
1. Neuroimmune													
IL-6	**↑**	**↑**	**↑**	**↑**	**↑**	**↑**	ns	ns	**↓**	-	-	ns	**↓**
TNF-alpha	-	-	+/−TG and +/+TG **↓**				overall ns			-	-	-	-
2. Signal transduction													
STAT3	**↑**	**↑**	+/−TG and +/+TG **↑**				overall ns			ns	ns	**↓**	ns
pSTAT3	**↑**	**↑**	**↑**	**↑**	**↑**	**↑**	ns	ns	**↓**	**↑**	**↓**	**↑**	**↓**
p42MAPK	ns	-	overall ns							-	-	ns	ns
pp42MAPK	ns	-	ns	ns	ns	ns	**↓**	ns	ns	-	-	ns	**↓**
p44MAPK	ns	-	overall ns							-	-	ns	ns
pp44MAPK	ns	-	overall ns							-	-	ns	**↓**

**↓** = significantly lower, **↑** = significantly higher, ns = relationship not significant, **-** = not determined, ^1^ data from [30,50,66], ^2^ data from [30], ^3^ data from [27,30].

**Table 4:** Summary of alcohol exposure/abstinence-induced changes in mean levels of hippocampal or cerebellar proteins.

Protein	Alcohol-Naïve ^1^		Alcohol-Exposed, Abstinent 3–8 Days						CIE, Abstinent 24 h			
	Hippocampus		Hippocampus			Hippocampus 2BC-CIE vs. 2BC			Hippocampus ^2^		Cerebellum ^3^	
	+/−TG vs. WT	+/+TG vs. WT	+/−TG vs. WT	+/+TG vs. WT	+/+TG vs. +/− TG	WT	+/− TG	+/+ TG	WT vs. Naïve	+/− vs. Naïve	WT vs. Naïve	+/−TG vs. Naive
1. Inhibitory synaptic transmission												
GABA_A_R a-1	ns	ns	ns	**↑**	**↑**	overall ns			ns	ns	ns	ns
GABA_A_R a-5	**↑**	**↑**	ns	**↑**	ns	overall ns			**↓**	**↓**	**↓**	**↓**
GAD 65	ns	**↓**	overall ns			overall ns			ns	ns	ns	**↓**
GAD 67	ns	**↓**	ns	**↓**	**↓**	overall ns			ns	ns	ns	**↓**
VGAT	**↑**	**-**	**↑** M	**↑** F,M	**↑** F	overall ns			**↓**	**↓**	**↓**	**↓**
Gephyrin	**↓**	**↓**	ns	**↓**	ns	overall **↓**			ns	ns	**↓**	ns
2. Excitatory synaptic transmission												
GluR1	ns	ns	ns overall ns			overall ns			-	-	**↓**	ns
NR1	ns	ns	**↑**	ns	**↓**	overall ns			-	-	-	-
PSD95	ns	ns	overall ns			overall **↓**			-	-	-	-

**↓** = significantly lower, **↑** = significantly higher, ns = relationship not significant, **-** = not determined, ^1^ data from [30,50,66], ^2^ data from [30], ^3^ data from [27,81].

## Data Availability

All data are available from the corresponding author upon a reasonable request.

## Supplementary Materials

The following supporting information can be downloaded at: https://www.mdpi.com/article/10.3390/cells12182306/s1. Complete Western blots, from which the sample Western blots for individual proteins shown in Figure 4 and Figure 5 were obtained, are available in the Supplementary Materials.
