# Supplementary figures and images for: Impact of Elevated Brain IL-6 in Transgenic Mice on the Behavioral and Neurochemical Consequences of Chronic Alcohol Exposure
Source: Cells. 2023 Sep 19;12(18):2306. doi: 10.3390/cells12182306 (PMC10527024; doi:10.3390/cells12182306)

Figure S1

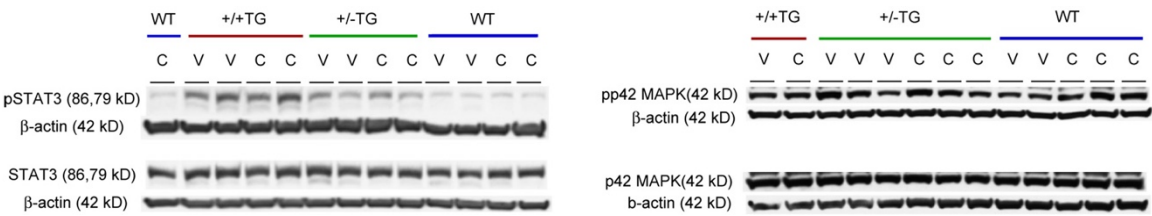

Figure S2

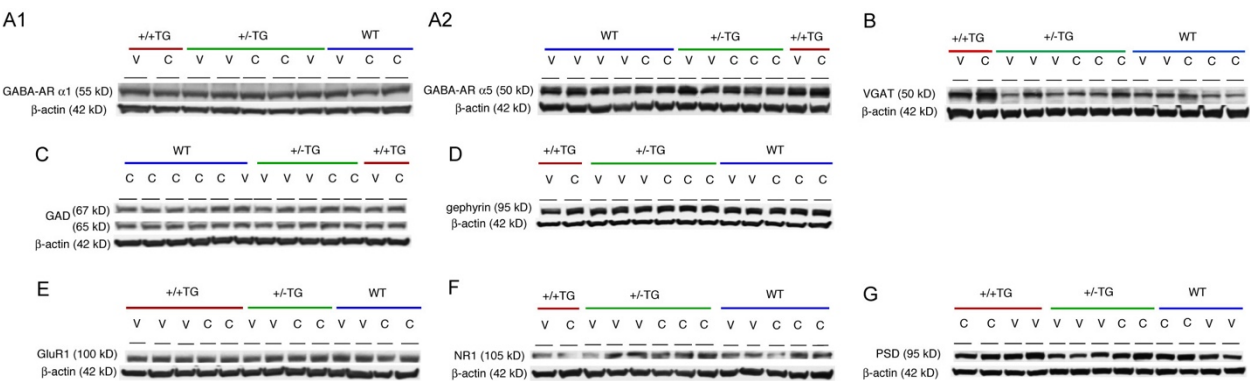

Supplement: Supplementary file 1 [file cells-12-02306-s001.zip › cells-2366637-supplementary.pdf]
